# Beyond "medical tourism": Canadian companies marketing medical travel

**DOI:** 10.1186/1744-8603-8-16

**Published:** 2012-06-15

**Authors:** Leigh Turner

**Affiliations:** 1 Center for Bioethics, School of Public Health, College of Pharmacy, University of Minnesota, Minneapolis, MN, USA

**Keywords:** Medical tourism, Canada, Transnational healthcare, Globalization, Websites

## Abstract

**Background:**

Despite having access to medically necessary care available through publicly funded provincial health care systems, some Canadians travel for treatment provided at international medical facilities as well as for-profit clinics found in several Canadian provinces. Canadians travel abroad for orthopaedic surgery, bariatric surgery, ophthalmologic surgery, stem cell injections, “Liberation therapy” for multiple sclerosis, and additional interventions. Both responding to public interest in medical travel and playing an important part in promoting the notion of a global marketplace for health services, many Canadian companies market medical travel.

**Methods:**

Research began with the goal of locating all medical tourism companies based in Canada. Various strategies were used to find such businesses. During the search process it became apparent that many Canadian business promoting medical travel are not medical tourism companies. To the contrary, numerous types of businesses promote medical travel. Once businesses promoting medical travel were identified, content analysis was used to extract information from company websites. Company websites were analyzed to establish: 1) where in Canada these businesses are located; 2) the destination countries and health care facilities that they market; 3) the medical procedures they promote; 4) core marketing messages; and 5) whether businesses market air travel, hotel accommodations, and holiday tours in addition to medical procedures.

**Results:**

Searches conducted from 2006 to 2011 resulted in identification of thirty-five Canadian businesses currently marketing various kinds of medical travel. The research project began with what seemed to be the straightforward goal of establishing how many medical tourism companies are based in Canada. Refinement of categories resulted in the identification of eighteen businesses fitting the category of what most researchers would identify as medical tourism companies. Seven other businesses market regional, cross-border health services available in the United States and intranational travel to clinics in Canada. In contrast to medical tourism companies, they do not market holiday tours in addition to medical care. Two companies occupy a narrow market niche and promote testing for CCSVI and “Liberation therapy” for multiple sclerosis. Three additional companies offer bariatric surgery and cosmetic surgery at facilities in Mexico. Four businesses offer health insurance products intended to cover the cost of obtaining privately financed health care in the U.S. These businesses also help their clients arrange treatment beyond Canada’s borders. Finally, one medical travel company based in Canada markets health services primarily to U.S. citizens.

**Conclusions:**

This article uses content analysis of websites of Canadian companies marketing medical travel to provide insight into Canada’s medical travel industry. The article reveals a complex marketplace with different types of companies taking distinct approaches to marketing medical travel.

## Background

In 2006 I began studying what many journalists and health researchers label “medical tourism”. A Canadian citizen, I started my research with particular interest in studying medical tourism companies based in Canada. I wanted to better understand why some Canadians seek medical procedures outside Canada. In addition, from the start of my research I examined ethical issues related to medical travel and the emergence of a global marketplace in health services [1-4]. As of 2011 I have identified sixty-three businesses that have head offices or affiliate offices in Canada and market health care at medical facilities based outside Canada. Of this total, twenty-eight companies are no longer operational. The remaining thirty-five companies continue marketing health care delivered at international facilities.

To provide insight into Canada’s medical travel industry, I use content analysis of company websites to study the many different businesses that are based in Canada and market access to health care provided at international destinations as well as in for-profit clinics in Canada. Before conducting content analysis of medical travel company websites I first had to identify all companies that are based in Canada and market medical travel to international health care facilities. From 2006 to 2011 I developed a database of Canadian businesses promoting medical travel. I began with the assumption that I would identify and describe Canadian medical tourism companies. However, as I studied various businesses, I noticed important differences in the types of companies marketing transnational and intranational health care.

Some Canadian companies market health care in such countries as Costa Rica, India, and Thailand; they also offer holiday tours and stays at resorts. These companies typically promise access to treatment at distant health care facilities, offer to arrange air travel and hotel accommodations, list different medical procedures, transfer medical records, and promote “all-inclusive” medical tourism packages ensuring access to affordable, timely, and high-quality care. These businesses fit the model of what most researchers characterize as medical tourism companies.

Other businesses in Canada promote medical travel but do not send their clients to distant international destinations. Rather, these companies promote regional, cross-border travel to hospitals and clinics in the United States. They also market intranational travel to for-profit clinics within Canada. There is a long history of cross-border medical travel between Canada and the United States; some companies attempt to facilitate such travel [5-8]. In contrast to many medical tourism companies, these companies are more focused on facilitating prompt access to medical procedures than combining health care with holidays.

In addition to identifying medical tourism companies and cross-border medical travel facilitators, I found numerous businesses occupying specific niches within the medical travel industry. For example, I found companies that specialize in coordinating access to testing for Chronic Cerebrospinal Venous Insufficiency (CCSVI) and “Liberation Therapy” for multiple sclerosis [9]. Other businesses market access to bariatric surgery and cosmetic surgery performed at international destinations. Also, I found Canadian businesses that market insurance products intended to promote access to health care facilities in the U.S [10,11]. These businesses offer critical illness insurance plans and other insurance products and also help their clients coordinate care outside Canada. Finally, I found one company that is based in Canada and yet markets medical procedures predominantly to U.S. citizens. I began my research by looking for Canadian medical tourism companies and in time discovered a marketplace considerably more complicated than I anticipated. I have tried to describe these different businesses and sort them into distinct categories.

To transform into a manageable endeavor analysis of such a large number of companies, I gathered data on basic features of these businesses. First, I identified companies and established where they are located within Canada. Second, I recorded destination nations and, where noted, particular health care facilities promoted by medical travel facilitators. Third, I identified the medical procedures and medical specialties businesses market. Fourth, I extracted core marketing messages of medical travel companies. Fifth, I analyzed company websites to determine whether businesses offer to book travel, make hotel reservations, and coordinate tours and side trips in destination nations.

## Methods

### Development of a database of medical travel companies located in Canada

Before conducting content analysis of company websites I first developed a database of businesses marketing medical travel. Numerous methods were used to build the database. From 2006 to July 2011, I conducted repeated Internet searches to identify medical travel companies with head offices or affiliate offices in Canada. In total I identified thirty-five companies currently promoting medical travel beyond Canada’s borders and 28 companies that have exited the marketplace for promoting transnational health care.

When conducting Internet searches I used such phrases as “medical tourism Canada”, “medical tourism company Canada”, medical tourism agency Canada” medical tourism facilitator Canada”, “medical tourism broker Canada”, “medical tourist Canada”, and “cross-border healthcare Canada”. I also used these terms when searching for newspaper articles describing Canadian medical travel companies. Searches for newspaper articles were conducted using Google News Canada and ProQuest Newsstand. Newspaper reports of medical tourism companies assisted with tracking the development of Canadian businesses marketing medical travel. Use of Google Alerts complemented searches for relevant newspaper articles. Google Alerts were created for such phrases as “medical tourism Canada”, “stem cell tourism Canada”, “transplant tourism Canada”, and “global health care Canada”. Industry Canada operates a website that can be searched to identify federally incorporated Canadian businesses [12]. Searches of this database revealed the identities of several Canadian medical tourism companies. In addition, I was able to identify additional businesses and assess the comprehensiveness of the database by discovering brief lists of Canadian medical travel companies [13-15]. Continuing my search for Canadian companies involved in promoting medical travel, in November 2009 I traveled to Toronto, Ontario and attended an exhibition and conference on medical tourism [16]. In short, multiple search strategies were used in an effort to find Canadian businesses engaged in marketing medical travel.

Phone calls and emails were used to establish whether companies continue to function. Companies were deemed to have exited the marketplace if they had expired websites, non-functioning email accounts and disconnected phone service, or seven phone calls and/or emails failed to elicit a response. Companies were deemed operational if respondents reported that the companies remain in business.

### Content analysis of websites of medical travel companies

Once medical travel companies were identified, content analysis was used to study the websites of these businesses [17]. Content analysis was performed by analyzing printed versions of company websites. There were five main elements to content analysis of company websites. First, content analysis was used to identify the province and city or town that company websites indicate as their primary address within Canada. Where affiliate offices within or outside Canada were noted, I recorded these locations as secondary sites. Acquiring this information makes it possible to establish where across Canada medical travel companies are based. Medical travel companies have, in effect, two “locations”. In some respects they are internet-based companies that occupy virtual space [18,19]. Acknowledging the powerful role of the Internet as a tool for marketing transnational health care, the geographic location of medical travel companies matters. In Canada, medical travel companies are bound by the laws and regulations of both the provinces in which they are based and by federal law. For the purpose of enforcing laws and regulations, the location of companies is consequential [20].

Second, I used content analysis to document countries and, where listed, the specific health care facilities that medical travel companies identify as potential destination sites for their clients. Some company websites list a single destination for prospective customers. Other companies provide lengthy lists of possible destinations for health care. Websites were analyzed for the purpose of trying to understand where Canadian medical travel companies offer to send their clients.

Third, I used content analysis to document medical procedures and/or medical specialties marketed by medical travel companies. Some company websites list such specialties as cardiology and orthopaedics. Other company websites provide lengthy lists of particular medical procedures. Yet other websites list both clinical specialties and particular medical interventions. This topic was addressed to establish the types of health services medical travel companies market.

Fourth, I reviewed company websites for core marketing statements. These statements were found on home pages of medical travel companies, in mission statements, and on pages labeled with such tags as “About Us” and “What We Do”. Marketing messages were then summarized to indicate whether companies emphasize such messages as affordable access to care, access to timely health services, access to high-quality care, and access to care in exotic destinations.

Fifth, content analysis was used to establish whether medical travel companies market air travel, book hotel reservations, and arrange side trips to local tourist sites and other holiday excursions in addition to marketing health services. This subject was addressed to explore how many medical travel companies promote tourism and travel-related services in addition to health care.

Detailed information was recorded for each category of analysis. Information extracted through content analysis was fact-checked and feedback was solicited from two senior colleagues. Readers can assess the quality of content analysis by comparing it to the primary data of medical travel company websites. All company names are disclosed, website addresses are provided, and company websites are electronically archived.

Following the initial round of content analysis, phone calls, emails, Internet searches and searches of an Industry Canada database were used to distinguish operational medical travel companies from businesses that have exited the marketplace for transnational health care. Companies with expired websites, non-functional and non-responding email addresses, and discontinued phone numbers were deemed to have exited the marketplace. Businesses that have exited the marketplace are the subject of a companion article. This article describes and analyzes operational Canadian medical travel companies.

## Results

### Types of medical travel companies

I began studying Canada’s medical travel industry with what I assumed was a straightforward plan to identify all medical tourism companies based in Canada. During the process of trying to establish how many businesses constitute Canada’s medical tourism industry, it became apparent that companies operate according to somewhat different business models. Of the thirty-five companies involved in coordinating medical travel, eighteen Canadian companies fall within the category of what most health researchers regard as medical tourism companies [21-24]. These companies promote health services provided in such international destinations as Barbados, Costa Rica, India, Mexico, and Thailand. Seven additional companies share much in common with the eighteen medical tourism companies promoting medical travel to distant locations. However, instead of marketing health services in such distant countries as India or Mexico they restrict themselves to promoting regional, cross-border travel to the United States as well as private clinics within Canada. These companies, could, with some justification, be included in the category of medical tourism companies. However, it is helpful to note that some medical travel companies promote regional, cross-border travel, as well as intranational travel within Canada, instead of marketing travel to distant international health care facilities. These companies emphasize timely access to care rather than promoting holiday excursions. Two businesses promote medical travel but restrict themselves to marketing testing for CCSVI and “Liberation therapy” for multiple sclerosis. Three additional businesses advertise bariatric surgery and cosmetic surgery performed at sites in Mexico. Four businesses market private health insurance products that permit Canadian citizens to obtain access to health care in the U.S. and Canada. These companies also help arrange care at medical facilities in the U.S. and private Canadian clinics. Finally, one medical travel company markets health services primarily to uninsured and underinsured U.S. citizens. Medical travel companies can be sorted into different categories even though they all promote transnational health care. They occupy distinct market niches, promote different kinds of medical procedures, and offer different kinds of services to prospective clients. This article identifies different types of companies marketing medical travel and does not attempt to use “medical tourism” as a catch-all term for describing every business involved in promoting transnational and intranational medical travel [25].

### Locations of medical travel companies

Of the eighteen medical tourism companies, five are based in British Columbia, four are located in Alberta, one is based in Saskatchewan, one is in Manitoba, four are in Ontario, and three are based in Quebec.

Seven companies market cross-border, regional medical travel to the U.S. as well as intranational medical travel within Canada, Two of these businesses are situated in British Columbia, four are based in Ontario, and one is located in Quebec.

The two companies marketing diagnostic testing for CCSVI and Liberation Therapy for multiple sclerosis are based in Manitoba and Ontario.

Three companies advertise out-of-country bariatric surgery and cosmetic surgery. One business is located in Alberta. The remaining two companies are based in Saskatchewan.

Four businesses market private health insurance products that enable access to U.S. health care facilities. Two of these companies are located in Alberta, one is based in Ontario, and one is located in Quebec. In addition to offering insurance products these companies help their clients arrange care in the U.S.

The lone Canadian medical travel company marketing primarily to U.S. citizens is located in British Columbia.

Some medical travel companies based in Canada identify affiliate offices or company representatives located outside Canada. Where company websites note such information I have recorded where company representatives situated outside Canada are based. Additional file 1 lists company names, identifies where in Canada these businesses are located, and indicates affiliates and company representatives in those instances where companies had offices or agents situated outside Canada.

### Destination countries and health care facilities

Of the eighteen medical tourism companies promoting global medical travel, seven marketed one country as a health care destination, two businesses marketed three health care destinations, three companies marketed five countries as destinations, one company marketed six destination nations, two companies marketed eight destination nations, one company marketed nine destination nations, one business marketed twelve destination nations, and one business listed thirty potential destination nations.

In total, the eighteen medical tourism companies promoting global medical travel list thirty-eight different destination nations. Eleven companies list India as a potential medical destination, nine companies list Coast Rica, seven companies list Thailand, seven list Mexico, six companies list the U.S., five companies list Singapore, five companies list Canada, three companies list South Africa, three companies list Brazil, three companies list Malaysia, three companies list Turkey, three companies list Barbados, two companies list Italy, two list Cuba, two list the Dominican Republic, two list Poland, two list Argentina, two list Panama, two list Colombia, two list Israel, and two list the United Arab Emirates (with one company indicating Dubai as a potential destination and one company identifying Abu Dhabi as a possible destination site). El Salvador, United Kingdom, Ukraine, Switzerland, Spain, South Korea, Ecuador, France, Austria, Bahamas, Belgium, Bulgaria, Germany, Hungary, Jordan, Lebanon, and Peru are all listed by a single medical tourism company. The list of possible destination nations, and the number of companies that mention them, reveals the variety of destinations marketed by medical tourism companies based in Canada as well as the most common destination nations promoted by Canadian medical tourism companies.

Of the seven companies marketing cross-border, regional travel to the U.S. and intranational travel to clinics within Canada, six companies indicate that tests and procedures can be obtained in both countries and one business restricts itself to sending clients to U.S. medical facilities. The lone company marketing medical travel primarily for diagnostic imaging is among the businesses offering access to health services in both the U.S. and Canada.

Of the two companies marketing medical travel for “CCSVI testing” and “Liberation therapy”, one business marketed diagnostic imaging in the U.S. and procedures in India. The other company markets both tests and procedures in India.

Three businesses market medical travel for bariatric surgery and cosmetic surgery. All of these companies advertise surgery performed in Mexico.

Four businesses market private insurance plans that enable access to health care in the United States. Three of the businesses note the possibility of obtaining some types of tests and treatments at medical facilities in Canada. One insurance program is restricted to enabling access to tests and procedures at three Mayo Clinic sites based in the U.S.

Finally, one medical travel company based in Canada targets U.S. customers rather than Canadians interested in travelling for medical care. This business markets access to medical procedures available in eight U.S. states as well as facilities in Canada.

It is important to make a distinction between how medical travel is marketed on company websites and actual practices of Canadians using services of medical travel businesses when going abroad for health care. When medical travel companies list various countries as potential destinations for their clients, it should not be assumed that Canadians have contracted with these companies and travelled to listed destinations. It is conceivable that clients of these businesses have never selected some listed countries. It is also possible that some nations are listed even though companies have no established relationships with health care providers in these countries. Acknowledging the possibility of a gap between how medical travel is marketed and behaviour of medical travelers, analyzing websites of Canadian medical travel companies provides insights into how these businesses attempt to market health services to prospective clients. Additional file 1 identifies locations of destination facilities marketed on company websites.

### Types of advertised medical procedures

Most of the 18 medical tourism companies in Canada offer comprehensive baskets of health services. However, some companies take niche positions by advertising restricted range of medical interventions. For heuristic purposes, medical travel companies can be classified as “generalist”, “specialist”, and “intermediate” firms. The former category includes businesses offering, for example, orthopaedic procedures, infertility treatments, cosmetic surgery, cardiac care, ophthalmology procedures, alternative medicine, and dental surgery. A company limited to marketing cosmetic surgery procedures can serve as an example of a specialist medical tourism company. Of the 18 medical tourism companies promoting health care at global destinations, 14 businesses operate as generalist medical travel firms. Two companies are specialist firms. One of them specializes in cosmetic surgery. The second firm predominantly markets procedures related to administration of human umbilical cord blood cells. Two businesses fall between these poles and can be classified as having “intermediate” marketing models. Of these latter businesses, one company emphasizes in its marketing claims access to cosmetic surgery but its list of procedures includes cosmetic surgery, dental surgery, bariatric surgery, gender-reassignment surgery, and non-surgical interventions. Another firm claims that it primarily markets alternative health care but also advertises orthopaedic surgery and reconstructive surgery.

Of the seven companies marketing cross-border, regional medical travel to health care facilities in the United States as well as private clinics in Canada, four companies can be classified as generalist firms. Of the remaining three businesses, one company promotes access to many different kinds of care but places particular emphasis upon diagnostics and arranging second opinions, one company offers comprehensive services but emphasizes preventive medicine and access to diagnostic tests, and one business specializes in marketing diagnostic imaging.

Of the remaining medical travel companies, two businesses market testing for CCSVI and “Liberation Therapy” for MS, and three businesses predominantly market bariatric surgery and cosmetic surgery. Both of the latter companies emphasize that they focus upon providing weight-loss treatments. These companies can all be classified as “specialist” medical travel firms.

Four companies market private health insurance plans enabling access to out-of-province and out-of country health care. These businesses offer insurance products that cover range of medical interventions. However, since their main product related to medical travel is related to selling critical illness insurance that can be used to cover cost of care at private medical facilities, they can be classified as specialist firms.

Finally, one medical travel company that is based in Canada markets health services mainly to clients from outside Canada. This company operates with a generalist model and offers such health services as cardiac surgery, general surgery, orthopedic surgery, neurosurgery, High Intensity Focused Ultrasound for prostate cancer, and additional medical interventions.

In summary, of thirty-five companies marketing medical travel, nineteen businesses operate using a generalist model based upon advertisement of many different kinds of medical interventions or insurance products and customer service intended to promote access to health care. Additional file 1 lists health services and medical specialties promoted by Canadian medical travel companies.

### Core marketing messages of medical travel companies

Most medical tourism companies promoting travel to global destinations use core marketing messages that emphasize access to affordable, timely, and high quality care. Of eighteen medical tourism companies marketing travel to global destinations, sixteen emphasize affordability of health care at international medical facilities. Access to timely health care is marketed by fifteen companies. All eighteen businesses market access to high-quality health care. Three businesses note that medical interventions can be obtained in exotic settings that appeal to tourists.

Of the seven businesses marketing regional, cross-border medical travel to the U.S. as well as intranational medical travel within Canada, six companies emphasize affordability of care. Six businesses also market timely access to treatment. Four companies emphasize the high-quality of care available at the destinations they promote.

Two companies market testing for CCSVI and Liberation Therapy. Both companies emphasize affordability and high-quality of marketed procedures; one business promotes timely access to treatment.

All three companies marketing bariatric surgery at facilities outside Canada emphasize comprehensive access to weight loss interventions. Their core marketing messages emphasize access to bariatric surgery and identify the variety of weight loss programs they offer. Their lists of specific procedures include cosmetic surgery.

Four businesses market insurance products that enable access to health care outside Canada or at private clinics within Canada. Three companies emphasize affordable access to care. All four companies promote timely access to health care and access to treatment at high-quality healthcare facilities.

Finally, the one medical travel company targeting non-residents of Canada promotes access to affordable, timely, and high-quality medical interventions.

There are variations in the types of themes companies use when marketing medical travel. However, messages concerning affordability of care, timely access to treatment, and access to high-quality medical interventions are common. Some companies use all three selling points to market health services at international destinations. Other businesses emphasize one or two key features of the health services they promote. Additional file 2 summarizes medical travel companies’ core marketing messages.

### “Tourism” component of medical travel

Medical travel companies typically emphasize affordability of care, timely access to care, and quality of care rather than tourism-related activities in their core marketing messages. Some companies note that they offer access to care in “exotic settings”. However, incorporating rhetoric concerning holiday-going and tourism activities into core marketing messages is atypical. Acknowledging that medical travel companies place greater emphasis on advertising medical interventions than promoting tourism, many businesses nonetheless market services associated with travel and tourism. Of the eighteen companies marketing medical tourism at global health care destinations, thirteen offer to coordinate travel arrangements, seventeen advertise the service of booking hotel reservations or otherwise arranging accommodations for clients, and fourteen offer to organize tours to local attractions located near where medical procedures are provided.

Of the seven companies marketing cross-border regional travel to the U.S. and intranational travel within Canada, four offer the service of booking travel and three offer to make hotel reservations. The possibility of booking tours to local holiday destinations is not addressed by these company websites.

Two companies market testing for CCSVI and Liberation Therapy. Of these businesses, one offers to book travel, both offer to make hotel reservations, and one advertises tours.

Three companies market weight-loss surgery and cosmetic surgery at international destinations. All three businesses offer to book travel; two companies offer to make hotel reservations. None of the companies mentions organizing tours and side trips before or after medical care is provided to clients.

Of the four companies marketing insurance products enabling access to care in the United States as well as private clinics in Canada just one business clearly indicates that it books travel. None of the companies state whether they book hotel reservations and organize tours. These companies emphasize offering insurance products and ensuring that their clients can obtain timely access to care in the United States or Canada. They help arrange care at U.S. facilities but they do not organize logistics to the same extent as medical tourism companies and regional, cross-border medical travel companies. In addition, they do not promote tours and holiday excursions.

The one Canadian medical tourism company marketing care to U.S. citizens does not indicate whether it books travel, make hotel reservations, or organize tours and other holiday excursions. It facilitates medical procedures and does not advertise additional services.

In summary, there is considerable variation in the extent to which medical travel companies market coordination of air travel, hotel reservations, and holiday excursions in addition to promoting medical procedures. Some companies embrace the concept of medical tourism and combine marketing of medical procedures with holiday excursions in “exotic” locations. Other businesses focus exclusively on arranging medical care at international destinations and make no attempt to promote and organize travel, accommodations, and tours.

Additional file 2 identifies whether medical travel companies advertise travel arrangements, offer to organize hotel accommodations, and market tours and side trips in addition to marketing health services.

## Discussion

This article provides a content analysis of company websites of thirty-five Canadian businesses that market medical travel. Content analysis of company websites is a valuable tool for exploring what services companies promote and how they attempt to position themselves in the marketplace [26,27]. Drawing upon content analysis of websites of medical travel companies, it is reasonable to conclude that Canada now has many businesses marketing access to medical procedures that are paid for out-of-pocket, rather than covered by provincial health insurance plans, and provided at international medical facilities. At present, Canada has a limited private health care sector [28,29]. Provincial health care systems provide universal access to “medically necessary” health services; legislation restricts the types of medical procedures for which private clinics are allowed to charge patients [30,31]. Canada’s effort to provide publicly-funded, universal access to health care is based on an egalitarian philosophy in which all citizens have rights to medical care and personal wealth cannot be used to jump to the front of queues and obtain treatment [32]. Legislation limiting expansion of a for-profit, private health care sector in Canada appears to have resulted in the emergence of companies that serve as bridges or intermediaries to hospitals and clinics located outside Canada. Some of these businesses also market intranational travel to private, for-profit medical clinics located in British Columbia, Alberta, Quebec, and elsewhere. Medical travel companies promote access to an out-of-country private health care sector available to Canadian citizens with sufficient financial resources. Various insurance products provide Canadians with sufficient financial resources tools for gaining access to health care facilities in the U.S.

Presumably wanting to appeal to the largest possible number of prospective clients, most medical travel companies offer many different kinds of medical procedures. Though Canadian provincial health insurance systems provide universal access to care for medically necessary services, Canadians must sometimes wait long periods for elective medical procedures [33-35]. Some medical travel companies respond to treatment delays in Canada by marketing swift access to private health care at international facilities. Orthopedic procedures, ophthalmologic procedures, bariatric surgery, and other interventions that sometimes require lengthy waits for care in Canada are readily available within the private health care sectors of other countries. Many Canadian medical tourism companies sending their clients to such countries as India and Thailand, as well as medical travel companies promoting regional, cross-border travel to the United States, in their core marketing messages emphasize timely access to care.

Assurances about providing timely access to care can be found on the websites of numerous medical travel companies based in Canada. Claims about offering access to high-quality care are also widespread. Clients of medical travel companies presumably want not just fast access to treatment but also access to professional medical care. Company websites offer messages intended to reassure clients about treatment available at international hospitals and clinics.

There are some circumstances where provincial health insurance plans in Canada permit reimbursement of expenses incurred as a result of receiving out-of-country medical care. In Canadian provinces, coverage of out-of-country care for elective procedures typically requires, at minimum, recommendation by a Canadian physician and pre-approval by provincial health ministries [36]. In most instances individuals travelling abroad for elective medical procedures must pay out-of-pocket for treatment [37]. Perhaps for this reason, many medical travel companies in Canada emphasize affordability of the medical procedures they market.

Some companies specialize in offering medical interventions that have not undergone clinical trials or regulatory review and are not approved for patient care in Canada. For example, medical travel companies marketing access to stem cell injections and “Liberation Therapy” help their clients gain access to procedures that patients cannot obtain in Canada. From one perspective, offering such services promotes “patient choice” by giving clients access to procedures they wish to undergo. However, expansion of choice likely also exposes some Canadians to considerable risk by enabling them to gain access to medical interventions with unknown safety and efficacy profiles. To date, at least two Canadians with multiple sclerosis are reported to have died while undergoing “Liberation Therapy” at medical facilities located outside Canada [38-40].

## Limitations

Multiple search methods were used to identify Canadian medical travel companies. However, Canada’s marketplace for medical travel is quite turbulent. New companies emerge, old businesses disappear, companies that have marketed medical travel for years periodically repackage themselves, drop old marketing initiatives, and fashion new identities. Due to limitations in search strategies and transformations in the marketplace for medical travel, it is possible that this article has not examined websites of all medical travel companies based in Canada. In addition, given fluidity in the marketplace, some companies that were operational when they were contacted might subsequently have gone out of business.

Internet searches and searches of databases containing newspaper articles were conducted in English. Though I was able to identify medical travel companies based in Quebec, it is conceivable that some companies marketing in French or other languages were not identified when using different search strategies.

Content analysis is useful when attempting to describe what companies claim to do. It is not a useful tool for attempting to establish whether there is a gap between promotional claims and actual company practices. Companies can engage in misrepresentation, use misleading advertising, and even defraud their customers by marketing health services and then failing to deliver what they are contractually obligated to provide [41,42]. Interviews, participant observation, surveys, and tools drawn from investigative journalism can all provide insight into possible gaps between how companies use the internet to market themselves and how they function in practice. Future research will help address the extent to which marketing claims resemble or differ from business practices.

This study has an obvious geographic limitation. I examine medical travel companies based in Canada. Though methods used in this paper can be applied to the study of medical travel companies in other countries, I do not address medical travel companies based outside Canada. Indeed, the paper does not address all of the medical travel companies that Canadians might consult when deciding whether to seek care outside Canada [43]. Canadians considering going abroad for treatment are free to contact destination hospitals or select health care packages provided by medical travel companies based outside Canada. Studying medical travel companies within Canada sets reasonable limits for research, and I suspect many Canadians consider using Canadian medical travel businesses if they decide to use a facilitator to arrange transnational health care. However, it is important to note that companies based in the United States and elsewhere also market health services to Canadian citizens.

## Comparison with prior work

### Focus on Canada

U.S. medical tourism companies are the subject of numerous studies [44-46]. Medical tourism companies based in Canada have attracted some attention from researchers but there is limited analysis of company websites and little exploration of the many medical travel companies that do not neatly fit into the category of medical tourism businesses [47-49]. This article examines the medical travel industry of a country with a publicly funded system of universal access to health care. There are presumably some important differences between why Canadians go abroad for health care and why residents of the United States and other countries travel for medical interventions. Scholarly research examining medical travel will benefit from analysis of many different social, economic, and cultural contexts.

### Investigating “medical tourism companies” as a subset of medical travel businesses

To date, researchers appear particularly interested in studying companies that promote medical travel to India, Thailand, and other distant, “global” health care destinations. This article makes distinctions among different types of companies in Canada’s medical travel industry. Some companies market health services available in countries located far from Canada. Other companies promote regional, cross-border travel to the U.S. as well as intranational travel within Canada. Yet other businesses promote specific interventions such as bariatric surgery and “Liberation therapy”. Some companies market insurance products and help Canadian clients obtain health care in the U.S and private facilities in Canada. Studies that focus strictly on medical tourism companies sending clients to distant nations risk missing or neglecting many of the businesses identified in this study. Businesses promoting medical travel are not reducible to medical tourism companies marketing health services and holidays available in faraway international locations. There is something to be gained from identifying differences within the medical travel industry and expanding the analytic lens to include both medical tourism companies and related businesses that promote medical travel but do not necessarily fit the standard model of medical tourism companies. Businesses promoting regional, cross-border care, weight loss, “Liberation therapy” and private insurance coupled with assistance arranging health care in the United States all market access to various types of medical travel.

### Canada’s medical travel industry as a bounded field

Several studies analyze particular facets of medical tourism company websites but do not use search strategies designed to identify companies based in particular countries. This study attempts to identify and analyze websites of all identified operational medical travel companies situated in Canada. Such an approach makes it possible to provide an overview of Canada’s medical travel industry.

### Disclosure of primary sources

This article identifies Canadian companies that market transnational health care, lists their websites, and electronically archives all company websites. Additional file 3 provides company names, notes their websites, and contains links to electronically archived copies of company websites. These steps mean that readers have access to all primary data used as the basis for this study. Access to primary data means that it is possible for readers to evaluate claims made in this paper, conduct novel analyses driven by research questions not addressed in this study, compare Canada’s medical travel industry with medical travel companies in other countries, and track over time changes in Canada’s medical travel industry. Identifying specific businesses and providing content analysis of company websites should facilitate further research into Canada’s medical travel industry and enable comparative, cross-national studies.

## Conclusions

Canada has a health care system providing what is often described as offering universal access to medically necessary care. Most citizens belong to publicly funded health insurance plans run by provinces. These plans are guided by the egalitarian philosophy that all citizens, regardless of social status and personal wealth, should have publicly funded access to “medically necessary” health services. In short, need for care rather than capacity to afford treatment is supposed to determine access to health care. Given that residents of Canada have universal access to medically necessary health services, individuals unfamiliar with health care in Canada might assume that with presumably limited demand few medical travel companies are likely to emerge in Canada. To the contrary, thirty-five businesses market medical travel.

Eighteen businesses market health care at such international destinations as Barbados, Costa Rica, India, and Thailand. Most of these companies offer wide range of health services. Many of these companies market air travel, hotel accommodations, and holiday excursions that can take place before or after receiving medical care. These companies take medical care and tourism and fuse them together to create a novel type of business enterprise.

Seven businesses market regional, cross-border health care. These companies advertise medical procedures that can be obtained in the United States and, in some instances, at private medical clinics in Canada. Though Canada has a private health care sector, it is limited in scope. In contrast, the U.S. has a large private health care sector in which it is possible to obtain ready access to care by paying out-of-pocket for treatment. Companies promoting regional, cross-border medical travel as well as intranational travel within Canada take advantage of the large private health care sector in the U.S. and the small but growing private health care sector in Canada [50-52]. In many respects they resemble the eighteen businesses identified as “standard” medical tourism companies. However, these businesses differ in terms of where they propose sending their clients. They also differ in the extent to which they promote tourism-and-travel-related activities in addition to marketing health services.

“Liberation therapy” and CCSVI as a diagnostic category for understanding multiple sclerosis receive extensive news media coverage in Canada. In response to public interest in Liberation Therapy, and perhaps also stoking interest in the procedure, two medical travel companies in Canada dedicate themselves exclusively to promoting access to testing for CCSVI and “Liberation Therapy”. The success of their business model is likely connected to whether “Liberation Therapy” continues to be seen by some Canadians with multiple sclerosis as potentially effective medical intervention [53,54].

Three companies market bariatric surgery performed at facilities based outside Canada. These companies are businesses that promote weight loss strategies; they are not exclusively dedicated to promoting medical travel for bariatric surgery. However, since these businesses send their clients to Mexico, I have categorized them among the different types of companies promoting medical travel. In Canada, patients must often face long waits for bariatric surgery. These companies have presumably responded to these delays by promoting prompt access to bariatric surgery at medical facilities beyond Canada’s borders.

Four businesses market critical illness insurance products that help Canadians have the financial resources they will need to obtain private health care within the United States or at private clinics within Canada. Across Canadian provinces there are numerous restrictions on private health insurance for medically necessary medical interventions [55]. These companies skirt these restrictions by providing insurance for health services delivered outside Canadian provincial health insurance plans. In addition to providing financial support for health care provided in the U.S. or Canada’s private health sector, these businesses help their clients arrange care at U.S. medical facilities. However, it is important to appreciate that in numerous respects these businesses differ from medical tourism companies. They do not, for example, market holiday excursions before or after treatment. Rather, they offer private health insurance products that can be used to cover expenses associated with obtaining out-of-pocket medical care.

Finally, one medical travel company based in Canada markets health services primarily to U.S. citizens. The U.S. has a large population of uninsured and underinsured individuals. This company, a sister company of a business that markets medical travel for Canadians, promotes to uninsured and underinsured Americans affordable access to health care within eight U.S. states.

Using content analysis of company websites, this article is meant to provide an overview of Canada’s medical travel industry. Content analysis of company websites reveals where these businesses are based, the health care destinations they promote to prospective clients, the health services they market, their core marketing messages, and whether companies market travel, hotel reservations, and tours in addition to advertising health services.

At present there is no resource that health researchers, clinicians, policy makers, journalists and other individuals can use as an accessible guide to analyzing both the general terrain of Canada’s medical travel industry and the particular features of specific businesses. This article contributes to scholarship by putting a guide into the hands of health researchers and other parties interested in further exploring intellectual territory that is fascinating, complex, little-known, worthy of further study, and raises numerous ethical, social, legal, and public health concerns.

## Supplementary Material

Additional file 1 Companies, Locations, Destinations and Advertised Medical Procedures. Click here for file

Additional file 2 Companies, Core Marketing Messages, Travel, and Tourism Services. Click here for file

Additional file 3 Websites and Electronically Archived Company Websites. Click here for file

## References

[B1] Turner L Medical tourism: Family medicine and international health-related travel Canadian Family Physician 2007 53 1639 1641 17934018 PMC2231416

[B2] Turner L Canadian Medicare and the Global Health Care Bazaar Policy Options 2007 73 77

[B3] Turner L “First World Health Care at Third World Prices”: Globalization, Bioethics and Medical Tourism BioSocieties 2007 2 303 325 10.1017/S1745855207005765

[B4] Turner L Quality in health care and globalization of health services: accreditation and regulatory oversight of medical tourism companies Int J Qual Health Care 2011 23 1 7 10.1093/intqhc/mzq078 21148210

[B5] Eggertson L Wait-list weary Canadians seek treatment abroad CMAJ 2006 174 1247 10.1503/cmaj.060219 16636318 PMC1435972

[B6] Korcok M Excess demand meets excess supply as referral companies link Canadian patients, US hospitals CMAJ 1997 157 767 770 9307566 PMC1228126

[B7] Katz S Verrilli D Barer M Canadians’ Use of U.S. Medical Services Health Aff 1998 17 225 235 10.1377/hlthaff.17.1.225 9455035

[B8] Katz S Cardiff K Pascali M Barer M Evans R Phantoms In the Snow: Canadians’ Use of Health Care Services in the United States Health Aff 2002 21 19 31 10.1377/hlthaff.21.3.19 12025983

[B9] Martin M MS clinic’s practices stir alarm Winnipeg Free Press 2011 Available from: http://www.winnipegfreepress.com/local/ms-clinics-practices-stir-alarm-113126744.html

[B10] Romaniuk R Manitobans can head to Mayo Clinic Winnipeg Sun 2011 Available from: http://www.winnipegsun.com/2011/07/13/mayo-clinic

[B11] Brodbeck T Medicare can’t fight market Winnipeg Sun 2011 Available from: http://www.winnipegsun.com/2011/07/13/medicare-cant-fight-market

[B12] Industry Canada. Corporations Canada Available at: https://www.ic.gc.ca/app/scr/cc/CorporationsCanada/fdrlCrpSrch.html;jsessionid=0000v9O3VPcCrmqiXhaZKPaOE2o:15dmaulj8?locale=en_CA

[B13] Floyd M Izenberg D Kelly B Medical services directory Maclean’s 2006 Available from: http://www.macleans.ca/science/health/article.jsp?content=20060501_126220_126220

[B14] Find Medical Travel Services Available at: http://www.findinghealthcare.ca/medical_tourism.html

[B15] Medical Tourism Facilitators Tourism Review.com 2009 50 52 Available at: http://www.tourism-review.com/fm956/m6.pdf

[B16] India medical tourism destination 2009: healthcare without borders. The first ever medical tourism exhibition and conference in Canada [Internet] 2009 Toronto: IMTD Available from: http://www.imtd2009.com/

[B17] Pope C Ziebland S Mays N Qualitative research in health care: Analysing qualitative data BMJ 2000 320 114 116 10.1136/bmj.320.7227.114 10625273 PMC1117368

[B18] Cormany D Baloglu S Medical travel facilitator websites: An exploratory study of web page contents and services offered to the prospective medical tourist Tourism Management 2011 32 709 716 10.1016/j.tourman.2010.02.008

[B19] Lunt N Hardey M Mannion R Nip, tuck and click: Medical tourism and the emergence of web-based health information The Open Medical Informatics Journal 2010 4 1 11 10.2174/1874431101004010001 20517465 PMC2874214

[B20] Puzic S Windsor police lay fraud charges against EcuMedical couple Windsor Star 2010 2010 2010

[B21] Mason A Wright KB Framing medical tourism: An examination of appeal, risk, convalescence, accreditation, and interactivity in medical tourism web sites Journal of Health Communication 2011 16 2 163 177 10.1080/10810730.2010.535105 21161812

[B22] Penney K Snyder J Crooks V Johnston R Risk communication and informed consent in the medical tourism industry: A thematic content analysis of Canadian broker websites BMC Medical Ethics 2011 12 17 10.1186/1472-6939-12-17 21943392 PMC3189886

[B23] York D Medical Tourism: the Trend Toward Outsourcing Medical Procedures to Foreign Countries Journal of Continuing Education in the Health Professions 2008 28 2 99 102 10.1002/chp.165 18521877

[B24] Connell J Medical tourism: Sea, sun, sand and…surgery Tourism Management 2006 27 1093 1100 10.1016/j.tourman.2005.11.005

[B25] Sobo EJ Medical travel: what it means, why it matters Medical Anthropology 2009 28 4 326 335 10.1080/01459740903303894 20182968

[B26] Sobo E Herlihy E Bicker M Selling medical travel to US patient-consumers: the cultural appeal of website marketing messages Anthropol Med 2011 18 119 136 10.1080/13648470.2010.525877 21563007

[B27] Lunt N Carrera P Systematic review of websites for prospective medical tourists Tourism Review 2011 66 57 67 10.1108/16605371111127224

[B28] Steinbrook R Private Health Care in Canada NEJM 2006 354 1661 1664 10.1056/NEJMp068064 16625005

[B29] Gordon M Berger P The Alluring Myth of Private Medicine CMAJ 1996 155 404 406 8752065 PMC1488043

[B30] Charles C Lomas J Giacomini M Medical necessity in Canadian health policy: four meanings and…a funeral? Milbank Quarterly 1997 75 365 394 10.1111/1468-0009.00060 9290634 PMC2751054

[B31] Flood C Archibald T The illegality of private health care in Canada CMAJ 2001 164 825 830 11276552 PMC80881

[B32] Gray C Visions of our health care future: Is a parallel private system the answer? CMAJ 1996 154 1084 1087 8625030 PMC1487568

[B33] Christou N Efthimiou E Bariatric surgery waiting times in Canada Can J. Surg 2009 52 229 234 19503668 PMC2689726

[B34] Legare J Li D Buth K How established wait time benchmarks significantly underestimate total wait times for cardiac surgery Can J. Cardiol 2010 26 E17 21 10.1016/S0828-282X(10)70337-8 20101361 PMC2827229

[B35] Snider M MacDonald S Pototschnik R Waiting times and patient perspectives for total hip and knee arthroplasty in rural and urban Ontario Can J 2005 48 355 360 PMC3211901 16248132

[B36] Lindberg M Risk J External Review of the Ontario Health Insurance Plan’s Out-of-Country Program 2007 1 18 Available from: http://www.health.gov.on.ca/english/public/pub/ministry_reports/out_of_country_ohip/out_of_country_ohip.pdf

[B37] Korcok M Ontario’s move to limit out-of-province health care spending pays off in big way CMAJ 1993 148 425 426 8347183 PMC1490474

[B38] Morrow A Man dies after controversial MS treatment, doctor says The Globe and Mail 2010 Available from: http://www.theglobeandmail.com/news/national/man-dies-after-controversial-ms-treatment-doctor-says/article1314585/

[B39] Alphonso C Death of MS patient fuels debate over new treatment The Globe and Mail 2010 Available from: http://www.theglobeandmail.com/news/national/death-of-ms-patient-fuels-debate-over-new-treatment/article1807127/

[B40] McClure M Woman with MS dies after treatment Winnipeg Free Press 2011 Available from: http://www.winnipegfreepress.com/canada/Woman-with-MS-dies-after-treatment--125269119.html

[B41] Puzic S Company that linked Canadian patients to U.S. health care folds The Windsor Star 2010

[B42] Puzic S Windsor police lay fraud charges against EcuMedical couple The Windsor Star 2010

[B43] Cohen T New York medical brokerage gains ground in Canada Times-Colonist 2011

[B44] Kumar S Breuing R Chahal R Globalization of Health Care Delivery in the United States through Medical Tourism Journal of Health Communication 2012 17 1 22 10.1080/10810730.2011.585699 22150120

[B45] Alleman BW Luger T Reisinger HS Martin R Horowitz MD Cram P Medical tourism services available to residents of the United States J Gen Intern Med 2010 26 492 497 21161425 10.1007/s11606-010-1582-8 PMC3077479

[B46] Shah KY Current status and emerging trends of international medical outsourcing in the United States—a qualitative study 2007 University of South Carolina doctoral dissertation Archived at: http://www.webcitation.org/5x9wiGYUu

[B47] Crooks V Snyder J Medical tourism: What Canadian family physicians need to know Canadian Family Physician 2011 57 5 527 529 21571709 PMC3093576

[B48] Snyder J Crooks V Adams K Kingsbury P Johnston R The patient’s physician one-step removed’: the evolving roles of medical tourism facilitators Journal of Medical ethics 2011 37 9 530 534 10.1136/jme.2011.042374 21478420

[B49] Johnston R Crooks V Adams K Snyder J Kingsbury P An Industry Perspective on Canadian Patients’ Involvement in Medical Tourism: Implications for Public Health BMC Public Health 2011 11 416 10.1186/1471-2458-11-416 21627830 PMC3117713

[B50] Mehra N Eroding Public Medicare: Lessons and Consequences of For-Profit Health Care Across Canada Eroding Public Medicare: Lessons and Consequences of For-Profit Health Care Across Canada 2008 6 1 169 Available from: http://www.web.net/ohc/Eroding%20Public%20Medicare.pdf

[B51] Revah G Bell C Shopping for High-Technology Treatment in Another Province Healthcare Policy 2007 2 49 55 19305732 PMC2585468

[B52] Silversides A Canada Health Act breaches are being ignored, pro-medicare groups charge CMAJ 2008 179 1112 1113 10.1503/cmaj.081690 19015555 PMC2582763

[B53] Blackwell T Is new MS research the real thing, or a media-driven frenzy National Post 2010 Available from: http://www.nationalpost.com/news/story.html?id=2475272

[B54] Weeks C An uncertain future lies ahead for Zamboni’s MS theory and its potential patients The Globe and Mail 2011 Available from: http://www.theglobeandmail.com/life/health-and-fitness/an-uncertain-future-lies-ahead-for-zambonis-ms-theory-and-its-potential-patients/article584981/

[B55] Dhalla I Private Health Insurance: An International Overview and Considerations for Canada Healthcare Quarterly 2007 10 89 96 18274001 10.12927/hcq.2013.19340

