# Supplementary material for: Beyond "medical tourism": Canadian companies marketing medical travel
Source: Global Health. 2012 Jun 15;8:16. doi: 10.1186/1744-8603-8-16 (PMC3503750; doi:10.1186/1744-8603-8-16)
Supplement: Additional file 1 — Companies, Locations, Destinations and Advertised Medical Procedures. [file 1744-8603-8-16-S1.doc]

**Additional file 3: Websites and Electronically Archived Company Websites**

**CANADIAN MEDICAL TOURISM COMPANIES**

| **Company, website & electronically archived website** |
| --- |
| Indus Health Tours  <http://www.indushealthtours.com/>  WebCite: <http://www.webcitation.org/60bX1jeSb> |
| Meditours  <http://www.meditours.org/>  WebCite:  <http://www.webcitation.org/60b1IMV0p> |
| Metamorphosis Medical Retreats  <http://www.metamedretreats.com/>  WebCite: <http://www.webcitation.org/60bNLD6mx> |
| Passport Medical  <http://passportmedical.com/>  WebCite: <http://www.webcitation.org/60bX9Pu5H> |
| Surgical Tourism Canada  <http://www.surgicaltourism.ca/>  WebCite: <http://www.webcitation.org/60bXTFslB> |
| Medical Concierge  <http://www.medicalconcierge.ca/public/main.html>  WebCite: <http://www.webcitation.org/60bXYoWPb> |
| Overseas Medical Services Canada Inc.  <http://www.livestemcells.net/>  WebCite: <http://www.webcitation.org/60bXdboo1> |
| Star Health Vacations  <http://starhealthvacations.com/shv/aboutus.aspx>  WebCite: <http://www.webcitation.org/60bYc1CbN> |
| SurgicalEscape  <http://surgicalescape.com/about.htm>  WebCite: <http://www.webcitation.org/60bYoVWBg> |
| Global Healthcare Connections Inc.  <http://www.globalhealthcareconnections.com/>  WebCite: <http://www.webcitation.org/60bYuinsy> |
| Choice Medical Services  <http://www.choicemedicalservices.com/>  WebCite: <http://www.webcitation.org/60bZ2sdDY> |
| Aalpha International Medical Tourism Organisers Inc. (AIMTO)  <http://www.aarexabroad.com/>  WebCite: <http://www.webcitation.org/60bZ9anjt> |
| Angels Global Healthcare (Angels Global Healthcare is a subdivision of Angels of Flight Canada Inc.)  <http://www.angelsglobalhealthcare.com/>  WebCite: <http://www.webcitation.org/60bZFjNjx>  <http://www.angelsofflightcanada.com/>  WebCite: <http://www.webcitation.org/60bZMxxxY> |
| CMN Inc. (Canadian Medical Network Inc.)  <http://www.canmednet.com/index.html>  WebCite: <http://www.webcitation.org/60bZTb2Su> |
| MEDLINK GLOBAL INC.  <http://medlinkglobal.com/contact_us.htm>  WebCite: <http://www.webcitation.org/60bZYLeZf> |
| Debson Medical Tourism  <http://www.debsonmedicaltourism.com/> |
| GoSculptura, Inc  <http://www.gosculptura.com/index.php> (and numerous other websites)  WebCite: <http://www.webcitation.org/60bZgF3mC> |
| Health Services International (Servimed) Inc./Services Sante  International (Servimed) Inc.  <http://www.hsi-ssi.com/en/index.html>  WebCite: <http://www.webcitation.org/60bZmK6sq> |

**CANADIAN COMPANIES MARKETING CROSS-BORDER MEDICAL TRAVEL TO U.S. AND INTRANATIONAL MEDICAL TRAVEL TO CANADIAN FACILITIES**

| **Company & website** |
| --- |
| OneWorld Medicare Inc.  <http://www.oneworldmedicare.com/>  WebCite: <http://www.webcitation.org/60ba397C5> |
| Timely Medical Alternatives Inc.  <http://www.timelymedical.ca/>  WebCite: <http://www.webcitation.org/60ba9Ll8Z> |
| Best Doctors Canada  <http://www.bestdoctors.com/canada/home.php>  WebCite: <http://www.webcitation.org/60baF9eLk> |
| International Health Care Providers Inc.  <http://ihcproviders.com/>  WebCite: <http://www.webcitation.org/60bWrw4Fj> |
| VIP Docs Inc.  <http://www.vipdocs.com/>  WebCite: <http://www.webcitation.org/60baLbMAZ> |
| VIP Health Options  <http://www.royalviphealth.com/>  WebCite: <http://www.webcitation.org/60baQqubk> |
| MedExtra  <http://www.medextra.com/>  WebCite: <http://www.webcitation.org/60bW1x5xx> |

**CANADIAN COMPANIES MARKETING MEDICAL TRAVEL FOR “CCSVI TESTING” & “LIBERATION THERAPY”**

| **Company & website** |
| --- |
| CCSVI Clinic  <http://ccsviclinic.ca/>  WebCite: <http://www.webcitation.org/60baX3dE8> |
| Liberation Gateway  <http://www.liberationgateway.com/>  WebCite: <http://www.webcitation.org/60bac0ofA> |

**CANADIAN COMPANIES MARKETING MEDICAL TRAVEL FOR WEIGHT LOSS SURGERY**

| **Company & website** |
| --- |
| Weight Loss For Eternity  <http://www.weightlossforeternity.ca/index.asp>  WebCite: <http://www.webcitation.org/60bagPF3X> |
| Weight Loss Forever  <http://www.weightlossforever.ca/>  WebCite: <http://www.webcitation.org/60bakml1s> |
| Weight No More Consulting  <http://www.weightnomoreconsulting.com/>  WebCite: <http://www.webcitation.org/60vDrtNBu> |

**CANADIAN COMPANIES MARKETING INSURANCE PRODUCTS ENABLING ACCESS TO CARE IN THE U.S.**

| **Company & website** |
| --- |
| Acure Health Corp.  <http://www.acurehealth.com/>  WebCite: <http://www.webcitation.org/60bapoDuZ>  Second website is:  <http://waitlistinsurance.com/>  WebCite:  <http://www.webcitation.org/60bau6DjX> |
| Canadian Equity Group Inc. sells and distributes MyCare Insurance Program; OneWorld Assist Inc. acts as case manager  <http://www.canequity.ca/>  WebCite: <http://www.webcitation.org/60bb16H6L>  <http://www.mycare.ca/mayoclinic/>  WebCite: <http://www.webcitation.org/60bb56JOq> |
| Right Choice Insurance Inc.  <http://www.rightchoiceinsurance.ca/>  Operates in partnership with MedExtra  WebCite: <http://www.webcitation.org/60bb9sgaJ> |
| Etfs Travel & Healthcare Solutions  <http://www.etfsinc.com/>  WebCite: <http://www.webcitation.org/60bbDxldd> |

**CANADIAN MEDICAL TRAVEL COMPANY MARKETING TO U.S. CITIZENS**

| **Company & website** |
| --- |
| North American Surgery Inc.  <http://northamericansurgery.com/>  WebCite: <http://www.webcitation.org/60bbIGiZQ> |
