# Supplementary material for: Beyond "medical tourism": Canadian companies marketing medical travel
Source: Global Health. 2012 Jun 15;8:16. doi: 10.1186/1744-8603-8-16 (PMC3503750; doi:10.1186/1744-8603-8-16)
Supplement: Additional file 2 — Companies, Core Marketing Messages, Travel, and Tourism Services. [file 1744-8603-8-16-S2.doc]

**Appendix One:**

**Medical Travel Companies, Locations, Destinations and Advertised Health Services**

**CANADIAN MEDICAL TOURISM COMPANIES**

| **Company** | **City & Province** | **Destination Facilities** | **Health Services Marketed** |
| --- | --- | --- | --- |
| Indus Health Tours | Vancouver, British Columbia; Indian Office: Tilak Nagar, Jaipur | Health care facilities, health resorts, and spas in India | Cardiology, orthopaedics, dental surgery, plastic and cosmetic surgery, minimally invasive surgery, ophthalmology, oncology, neurology, neural surgery, gastroenterology, urology, nephrology, gynecology, Ayurveda, meditation, Yoga, spa |
| Meditours | Kelowna, British Columbia | Western India Institute Neurosciences, Kolhapur, India; Jaslok Hospital & Research Centre, Mumbai, India | Orthopedic surgery for shoulder, hip, knee, elbow, and wrist, plastic surgery (breast implants, face lift, liposuction, tummy tuck), Liberation treatment for CCSVI, Thalamontomy treatment for Parkinsons, Stem cell treatment for hip Necrosis, hip resurfacing, knee replacements, treatment for dystonia, tattoo removal |
| Metamorphosis Medical Retreats | Vancouver, British Columbia | Bangkok Hospital Medical Center, Yanhee Hospital, and other facilities in Thailand | plastic & cosmetic surgery (including breast augmentation, liposuction, tummy tuck/abdominoplasty, rhinoplasty, breast lift, breast reduction, eyelid surgery, brow lift, forehead lift, buttock augmentation, neck lift, cheek implants, arm lifts, chin implants), dental surgery, bariatric surgery, gender reassignment surgery, non-surgical interventions (spa, teeth whitening, chemical peels, Botox, Lasik, scar and tattoo removal) |
| Passport Medical  Divisions of Passport Medical:  IVF Vacation Center and CCSVI Cancun | Vancouver, British Columbia  Vancouver,  British Columbia; U.S. Office: Beverly Hills, California | Costa Rica, El Salvador, Mexico (Cancun), Panama, South Africa  Argentina, Cancun (Mexico), Costa  Rica, India, Panama, South Africa, Ukraine | CCSVI Liberation Treatment, Dental procedures (fillings, crowns, veneers, implants, whitening), Cosmetic surgery (including breast implants, face lift, tummy tuck, rhinoplasty, liposuction), Orthopedic surgery (including knee replacement, hip replacement, hip resurfacing), IVF fertility treatments (including IVF with ICSI, IVF with egg donation, surrogacy including IVF), ophthalmology (including LASIK and cataract surgery), type 2 Diabetes treatment, weight loss surgery (including gastric bypass, gastric band, gastric sleeve, intragastric balloon), In Vitro Fertilization, IVF with Egg Donation abroad, Commercial surrogacy |
| Surgical Tourism Canada | Vancouver, British Columbia; Canadian Branch offices: Western Canada, Surrey, British Columbia, Eastern Canada, Toronto, Ontario; US Office, Seattle, Washington | Apollo Hospitals in Chennai, Delhi, Bangalore, Hyderabad; Apollo Bramwell Hospital in Mauritius, Fortis Healthcare hospitals in Chennai, Mumbai, Bangalore, and Delhi, India; Arizona Doppler Specialists in Tucson Arizona/Saguaro Surgical in Tucson, Arizona/Tucscon Medical Centre, Tucson, Arizona; Amerimed American Hospitals, Puerto Vallarta, Cabo San Lucas, Cancun, Mexico;  Soukya Holistic Health Centre, Bangalore, India;  St. Joseph Hospital, Bellingham, Washington, US; Gulf Diagnostic Centre, Abu Dhabi, United Arab Emirates; The Clinic, New Delhi, India | CCSVI/MS Testing & the Liberation Procedure, stem cell therapy, orthopedics/spine surgery, bariatric surgery, cosmetic surgery, dental procedures, cardiology, oncology, in vitro fertilization, neurosurgery, nephrology, private physician consultations/2^nd^ opinions, preventative health checks, diagnostic services, MRI services, PET scan services, wellness clinics for complementary and Ayurvedic medicine |
| Medical Concierge | Calgary, Alberta | The Mayo Clinic, Scottsdale, Arizona; USA; L’Alberto Della Regina Isabella Spa and Resort, Naples, Italy; Dubai International Private Hospital, Dubai, UAE; Willows Stream Spa, Banff, Canada; University of Texas MD Anderson Cancer Center, Houston, USA; Cleveland Hospital, Cleveland, USA; Johns Hopkins Hospital, Baltimore, USA; Children’s Hospital, Boston, USA; Duke Medical Center, Durham, USA; Mount Sinai Medical Center, New York, USA; Dubai Sports Hospital, Dubai, UAE; Gleneagles Hospital, Singapore; East Shore Hospital, Singapore | Diagnostic testing, surgical procedures, spa care, “posh prenatal” care, cataract surgery, other treatments |
| Overseas Medical Services Canada Inc. | Calgary, Alberta; Affiliate Offices: Portland, Oregon, U.S.; Loja, Ecuador; Saudi Arabia | Ecuador | Human Umbilical Cord Blood Cells, additional surgical procedures and therapies |
| Star Health Vacations | Edmonton, Alberta | Apollo Hospital, Chennai; Apollo Hospital, Hyderabad, Indraprastha Apollo Hospital, New Delhi; Shroff Eye Hospital, Mumbia; Wockhardt Hospital, Mumbia; all in India | Joint surgery, face treatments, breast surgery, body plastic surgery, skin treatment, male plastic surgery, plastic and reconstructive surgery, spa treatments, energy healing, hypnosis, gem therapy, guided imagery |
| SurgicalEscape | Calgary, Alberta | Costa Rica; Barbados; Los Cabos, Mexico | Cosmetic and plastic surgery, bariatric surgery, orthopedics, dentistry, ophthalmology, medical check ups, dermatology, IVF |
| Global Healthcare Connections Inc. | Saskatoon, Saskatchewan | Canada, Costa Rica, Dominican Republic, India, Mexico, Singapore, Thailand, United States | Dental, Cardiology-Heart Care, Bariatric-Weight loss surgery Cosmetic non-surgical, Cosmetic surgical, Diagnostic Imaging-Health assessment, Hair transplant restoration, MS Treatment (CCSVI-Liberation Treatment, MS screening, doppler ultrasound), Oncology-Cancer care, Orthopaedics and Spine, Opthalmology, Stem Cell and Regenerative Treatments, medical second opinions |
| Choice Medical Services | Winnipeg, Manitoba | Canada, Costa Rica, Cuba | Cosmetic and plastic surgery, diagnostic procedures, general procedures, women’s health, vascular surgery, men’s health, orthopedic surgery, obesity management, eye surgery, nose and throat surgery, gynecological surgery, neurosurgery, addition services, preventative checkup, internal medicine, dental procedures, neurology, cardiology |
| Aalpha International Medical Tourism Organisers Inc. (AIMTO) | Brampton, Ontario | Apollo Hospitals, Fortis Hospitals, Chettinad Health City, Nishtha Retina Centre; all facilities in India | Heart-cardiology, dermatology, cosmetology and plastic surgery, cancer treatments-oncology, dental, ENT, nephrology and urology, neurology and neurosurgery, obstetrics, gynecology and infertility treatments, ophthalmology, orthopaedics and joint replacement, paediatrics, physical medicine and rehabilitation, preventive health check-up, psychiatry, Ayurveda |
| Angels Global Healthcare | Peterborough, Ontario | Barbados, Canada, Costa Rica, India, Israel, Mexico, Thailand, USA | Bariatric surgery, cardiology and cardiovascular procedures, cosmetic and reconstructive procedures, dental procedures, diagnostic and therapeutic procedures, general surgery, IVF and reproductive medicine, oncology (cancer) treatment, orthopedics and spine procedures, rehabilitation, wellness and executive check ups, other procedures |
| CMN Inc. (Canadian Medical Network Inc.) | Thornhill, Ontario | Argentina, Brazil, Costa Rica, France, India, Malaysia, Poland, Singapore, South Africa, Thailand, Turkey, U.S. | Gastric bypass, heart bypass, heart valve replacement, hip replacement, knee replacement, mastectomy, spinal fusion, additional tests and treatments |
| MEDLINK GLOBAL INC. | Toronto, Ontario; Affiliate offices in United Kingdom, United States, and Pakistan | Thailand, Costa Rica, India, Singapore, Malaysia, Turkey | joint Replacement surgery, cosmetic surgery, prevention and alternative medicine, weight loss surgery, ophthalmic procedures, cardiac surgery, reconstructive surgery, diagnostic imaging |
| Debson Medical Tourism | Verdun, Quebec and New York, US | Austria, Bahamas, Barbados, Belgium, Brazil, Bulgaria, Canada, Colombia, Costa Rica, Germany, Hungary, India, Israel, Italy, Jordan, Lebanon, Malaysia, Mexico, Panama, Peru, Singapore, South Africa, South Korea, Spain, Switzerland, Thailand, Turkey, Ukraine, United Kingdom, United States; 62 specified partnering treatment facilities identified | cancer, cosmetic, dental, eye, fertility, heart, orthopedics and spine, stem cell, transplantation, weight loss, wellness and spa |
| GoSculptura, Inc | Montreal, Quebec | Argentina, Brazil, Columbia, Costa Rica, Dominican Republic, India, Mexico, Poland, Thailand | Plastic surgery, gastric bypass, gastric banding, sleeve gastrectomy, liposuction, cosmetic dentistry, hair transplantation, ophthalmology surgery, dermatology, in vitro fertilization (IVF) |
| Health Services International (Servimed) Inc./Services Sante  International (Servimed) Inc. | Gabrielle d’Anneville, Quebec | Cuba (over 20 hospitals) | Retinitis pigmentosa, vitiligo and psoriasis, orthopedic surgery, dental care, hair loss, examinations and diagnostics, cosmetic surgery, diabetes, detoxification, diabetes, neurological rehabilitation, additional treatments |

**CANADIAN COMPANIES MARKETING CROSS-BORDER MEDICAL TRAVEL TO U.S.**

**AND INTRANATIONAL MEDICAL TRAVEL TO CANADIAN FACILITIES**

| **Company** | **City & Province** | **Destination Facilities** | **Health Services Marketed** |
| --- | --- | --- | --- |
| OneWorld Medicare Inc. | Richmond, British Columbia | United States and private clinics in Canada | hip replacements, knee replacements, MRI, CT, and other procedures |
| Timely Medical Alternatives Inc. | North Vancouver, British Columbia | Canada and U.S. (22 hospitals in 8 U.S. states): Arizona, Oklahoma, Montana, Kansas, Nevada, South Dakota, Maine, Maryland | MRI, CT Scans, PET Scans, Ultrasounds, Echocardiogram, colonoscopy, gastroscopy, orthopedic surgery, neurosurgery, cardiac surgery, general surgery, knee replacement, gall bladder removal, angioplasty, cardiac bypass, arthroscopic shoulder surgery, spinal discectomy, weight loss surgery, hip replacement, cardiac ablation, and additional diagnostic procedures and treatments |
| Best Doctors Canada | Toronto, Ontario | Hospitals and clinics in Canada and the United States | Second opinions and review of treatment options, retesting of pathology, treatment at facilities in Canada and the United States |
| International Health Care Providers Inc. | Windsor, Ontario | Detroit Medical Center, Oakwood Imaging Center, Regional Medical Imaging, Sky Ridge Medical Center, Verdugo Hills Hospital, First Hill Diagnostic Imaging, Indiana Orthopaedic Hospital, Pacific Interventionalists, California, and additional facilities in the US | Orthopedic surgery, cardiology, cancer, consultation services, psychiatric care, occupational ophthalmology, ear, nose & throat, neurology, endocrinology, pediatric endocrinology, gastroenterology, diagnostics, preventative diagnostics, bariatrics, gynecology, vascular birthmarks, dermatology, sleep clinics, laproscopic robotic prostate cancer surgery, CCSVI testing and treatment |
| VIP Docs Inc. | Burlington, Ontario | Buffalo General Hospital, New York, diagnostic imaging facilities in Toronto, Ontario | MRI Scans, CAT Scans, PET Scans, CTA Scan, MRA Scan, MRCP Scan, TMJ MRI Scan, Ultrasound, Mammography, assessments, referrals to US physicians, surgery |
| VIP Health Options | Burlington, Ontario | Network of 10 clinics and health centres in Canada and 8 clinics and hospitals in the U.S. | MRI exams, Executive medicals, comprehensive health assessments, preventive health care, risk assessment, cancer care, and other diagnostic tests and treatments; critical illness insurance for out-of-country healthcare, second opinions |
| MedExtra | Saint-Laurent, Quebec | Diagnostic and treatment facilities in Canada and the U.S. | Diagnostic testing, second opinions, management of cancer treatments and other therapies, minimally invasive surgical options, major surgeries, diagnosis of unidentified ailments |

**CANADIAN COMPANIES MARKETING MEDICAL TRAVEL FOR “CCSVI TESTING” & “LIBERATION THERAPY”**

| **Company** | **City & Province** | **Destination Facilities** | **Health Services Marketed** |
| --- | --- | --- | --- |
| CCSVI Clinic | Winnipeg, Manitoba | Doppler Ultrasound Screening for “Chronic Cerebral Spinal Venous Insufficiency (CCSVI) performed at Kidney and Hyptertension Center, Aurora Medical Center, in Grand Forks, North Dakota and clinics in British Columbia; Venous angioplasty “Liberation Therapy” procedure performed at Noble Hospital, Pune, India | Doppler Ultrasound Screening for CCSVI; “Liberation Therapy” procedure for individuals with multiple sclerosis; “stem cell therapy” in combination with venous angioplasty procedure |
| Liberation Gateway | Waterloo, Ontario; U.S. Office: Detroit, Michigan | Jaipur Golden Hospital, New Delhi, India | CCSVI Imaging and Stenting |

**CANADIAN COMPANIES MARKETING MEDICAL TRAVEL FOR WEIGHT LOSS SURGERY**

| **Company** | **City & Province** | **Destination Facilities** | **Health Services Marketed** |
| --- | --- | --- | --- |
| Weight Loss For Eternity | Edmonton, Alberta | Mexico | Weight loss surgery, cosmetic surgery |
| Weight Loss Forever | Saskatoon, Saskatchewan; Affiliate Offices in Lethbridge, Alberta and Regina, Saskatchewan | Weight Loss Surgical Center in Tijuana, Mexico | Weight loss surgery, cosmetic surgery |
| Weight No More Consulting | Saskatoon, Saskatchewan | Tijuana, Mexico | Weight loss surgery, cosmetic surgery |

**CANADIAN COMPANIES MARKETING INSURANCE PRODUCTS ENABLING ACCESS TO CARE IN THE U.S.**

| **Company** | **City & Province** | **Destination Facilities** | **Health Services Marketed** |
| --- | --- | --- | --- |
| Acure Health Corp. | Calgary, Alberta | Hospitals and clinics in Canada and the United States | Critical illness insurance, health insurance providing access to MRI & CT scans, colonoscopy, heart bypass, angiography, angioplasty, heart valve repair, arthroscopic procedures, endoscopic procedures, prostate surgery, hernia, slipped disc, cataract surgery, specialist appointments in following specialties: orthopaedic, cardiology, general surgery, gastroenterology, ophthalmology, spine team, neurology, rheumatology, urology, ear, nose & throat |
| Canadian Equity Group Inc. sells and distributes MyCare Insurance Program | Calgary, Alberta | Mayo Clinic, Rochester Minnesota; Phoenix/Scottsdale, Arizona; Jacksonville, Florida | Health insurance providing access to medical reviews, additional diagnostic testing, access to treatment, and diagnostic consultations provided by Mayo Clinic physicians |
| Right Choice Insurance Inc. | Toronto, Ontario | Arranges care at medical facilities in Canada and the United States | Critical illness insurance provides cash payment, access to second opinions, and access to out-of-province and out-of country care |
| Etfs Travel & Healthcare Solutions | Head Office: Sherbrooke, Quebec; Additional Offices in Montreal, Quebec; Toronto, Ontario; Vancouver, British Columbia | Network of US and Canadian healthcare facilities | Health insurance product is intended to cover range of care including heart and heart surgery, cancer, digestive disorders, ear, nose and throat, endocrinology, gynecology, neurology, ophthalmology, orthopedics, pediatrics, respiratory, and urology |

**CANADIAN MEDICAL TRAVEL COMPANY MARKETING TO U.S. CITIZENS**

| **Company** | **City & Province** | **Destination Facilities** | **Health Services Marketed** |
| --- | --- | --- | --- |
| North American Surgery Inc. | Vancouver, British Columbia | Montana, South Dakota, Maine, Nevada, Arizona, Oklahoma, Maryland, Kansas; facilities in Canada | cardiac surgery, general surgery, orthopedic surgery, neurosurgery, women’s procedures, spinal surgery, High Intensity Focused Ultrasound (HIFU) treatment for prostate cancer, additional treatments |
