# Supplementary material for: Beyond "medical tourism": Canadian companies marketing medical travel
Source: Global Health. 2012 Jun 15;8:16. doi: 10.1186/1744-8603-8-16 (PMC3503750; doi:10.1186/1744-8603-8-16)
Supplement: Additional file 3 — Websites and Electronically Archived Company Websites. [file 1744-8603-8-16-S3.doc]

**Appendix Two:**

**Companies, Core Marketing Messages, Travel and Tourism Services**

**CANADIAN MEDICAL TOURISM COMPANIES**

| **Company** | **Core Marketing Message** | **Book Travel** | **Book Hotel** | **Book tours** |
| --- | --- | --- | --- | --- |
| Indus Health Tours | access to affordable, timely, and high-quality healthcare | Y | Y | Y |
| Meditours | access to affordable, timely, and high quality healthcare in exotic settings | Y | Y | Y |
| Metamorphosis Medical Retreats | access to affordable and high-quality cosmetic surgery in exotic settings | NA | Y | Y |
| Passport Medical | access to affordable, timely, and high-quality healthcare | N | Y | R |
| Surgical Tourism Canada | access to affordable, timely, and high-quality healthcare | Y | Y | NA |
| Medical Concierge | access to timely and high-quality healthcare | R | R | NA |
| Overseas Medical Services Canada Inc. | access to affordable, timely, and high-quality healthcare | Y | Y | Y |
| Star Health Vacations | access to affordable and high-quality healthcare | NA | Y | Y |
| SurgicalEscape | access to affordable, timely, and high-quality healthcare | Y | Y | Y |
| Global Healthcare Connections Inc. | access to affordable, timely, and high-quality healthcare | Y | Y | Y |
| Choice Medical Services | access to affordable, timely, and high-quality healthcare | Y | Y | Y |
| Aalpha International Medical Tourism Organisers Inc. (AIMTO) | access to affordable, timely, and high-quality healthcare | Y | Y | Y |
| Angels Global Healthcare | access to affordable, timely, and high-quality healthcare | Y | Y | Y |
| CMN Inc. (Canadian Medical Network Inc.) | access to affordable, timely, and high-quality healthcare | Y | Y | NA |
| MEDLINK GLOBAL INC. | access to affordable, timely, and high-quality healthcare | Y | Y | Y |
| Debson Medical Tourism | access to affordable, timely, and high-quality healthcare | Y | Y | Y |
| GoSculptura, Inc | access to affordable and high-quality plastic surgery and other procedures in exotic locations | Y | Y | Y |
| Health Services International (Servimed) Inc./Services Sante  International (Servimed) Inc. | access to timely and high-quality healthcare | R | Y | Y |

**CANADIAN COMPANIES MARKETING CROSS-BORDER MEDICAL TRAVEL TO U.S. AND INTRANATIONAL MEDICAL TRAVEL TO CANADIAN FACILITIES**

| **Company & website** | **Core Marketing Message** | **Book Travel** | **Book Hotel** | **Book Tours** |
| --- | --- | --- | --- | --- |
| OneWorld Medicare Inc. | access to affordable, timely and high-quality care and medical access insurance | Y | Y | NA |
| Timely Medical Alternatives Inc. | access to affordable and timely healthcare | NA | NA | NA |
| Best Doctors Canada | access to affordable and high-quality care | Y | Y | NA |
| International Health Care Providers Inc. | access to affordable, timely, and high-quality healthcare | Y | Y | NA |
| VIP Docs Inc. | access to timely healthcare | NA | R | NA |
| VIP Health Options | access to affordable, timely, and high quality healthcare; access to critical illness insurance | Y | NA | NA |
| MedExtra | access to affordable and timely care | NA | NA | NA |

**CANADIAN COMPANIES MARKETING MEDICAL TRAVEL**

**FOR “CCSVI TESTING” & “LIBERATION THERAPY”**

| **Company & website** | **Core Marketing Message** | **Book**  **Travel** | **Book**  **Hotel** | **Book Tours** |
| --- | --- | --- | --- | --- |
| CCSVI Clinic | access to affordable and high-quality testing for CCSVI and Liberation Therapy | Y | Y | Y |
| Liberation Gateway | access to affordable, timely and high-quality “Liberation Therapy” | N | Y | NA |

**CANADIAN COMPANIES MARKETING MEDICAL TRAVEL FOR WEIGHT LOSS SURGERY**

| **Company & Website** | **Core Marketing Message** | **Book Travel** | **Book Hotel** | **Book Tours** |
| --- | --- | --- | --- | --- |
| Weight Loss For Eternity | access to bariatric surgery and cosmetic surgery | Y | NA | NA |
| Weight Loss Forever | access to high-quality weight loss surgery and cosmetic surgery | Y | Y | NA |
| Weight No More Consulting | Access to high-quality bariatric surgery and cosmetic surgery | Y | Y | NA |

**CANADIAN COMPANIES MARKETING INSURANCE PRODUCTS ENABLING ACCESS TO CARE IN THE U.S.**

| **Company & website** | **Core Marketing Message** | **Book Travel** | **Book Hotel** | **Book Tours** |
| --- | --- | --- | --- | --- |
| Acure Health Corp. | Insurance program providing access to affordable, timely, and high-quality care | Y | NA | NA |
| Canadian Equity Group Inc. sells and distributes MyCare Insurance Program; OneWorld Assist Inc. acts as case manager | Insurance program providing access to affordable, timely, and high-quality care | NA | NA | N |
| Right Choice Insurance Inc. | Critical illness insurance providing access to affordable, timely, and high-quality healthcare in U.S. and select Canadian medical facilities | NA | NA | NA |
| Etfs Travel & Healthcare Solutions | Private health insurance permitting access to timely and high-quality healthcare in U.S. and select Canadian medical facilities | NA | NA | NA |

**CANADIAN MEDICAL TRAVEL COMPANY MARKETING TO U.S. CITIZENS**

| **Company & website** | **Core Marketing Message** | **Book Travel** | **Book**  **Hotel** | **Book Tours** |
| --- | --- | --- | --- | --- |
| North American Surgery Inc. | access to affordable, timely, and high-quality healthcare | NA | NA | NA |
